# ATF3 Plays a Key Role in Kdo_2_-Lipid A-Induced TLR4-Dependent Gene Expression via NF-κB Activation

**DOI:** 10.1371/journal.pone.0014181

**Published:** 2010-12-02

**Authors:** Eun-Young Kim, Hye Young Shin, Joo-Young Kim, Dong-Gun Kim, Yong-Min Choi, Hyuk-Kwon Kwon, Dong-Kwon Rhee, You-Sun Kim, Sangdun Choi

**Affiliations:** 1 Department of Molecular Science and Technology, Ajou University, Suwon, Korea; 2 Institute for Medical Sciences, Ajou University School of Medicine, Suwon, Korea; 3 School of Pharmacy, Sungkyunkwan University, Suwon, Korea; Universidade de São Paulo, Brazil

## Abstract

**Background:**

Activating transcription factor 3 (ATF3) is a negative regulator of proinflammatory cytokine expression in macrophages, and ATF3 deficient mice are more susceptible to endotoxic shock. This study addresses the role of ATF3 in the Kdo_2_-Lipid A-induced Toll-like receptor 4 (TLR4) signaling pathway in mouse embryonic fibroblasts (MEF). Kdo_2_-Lipid A upregulates ATF3 expression in wild type MEF cells and induces both nuclear factor kappa B (NF-κB) and c-Jun N-terminal kinase (JNK) activation via the TLR4 signaling pathway, while neither of these pathways is activated in ATF3-/- MEF cells. Interestingly, in contrast to Kdo_2_-Lipid A, the activation of both NF-κB and JNK by TNF-α was normal in ATF3-/- MEF cells.

**Methodology/Principal Findings:**

We found that several genes were dramatically upregulated in ATF3+/+ MEF cells in response to Kdo_2_-Lipid A treatment, while little difference was observed in the ATF3-/- MEF cells. However, we also found that the signal intensities of IκBζ in ATF3-/- MEF cells were substantially higher than those in wild type MEF cells upon microarray analyses, and upregulated IκBζ expression was detected in the cytosol fraction.

**Conclusions/Significance:**

Our findings indicate that ATF3 deficiency affects Kdo_2_-Lipid A-induced TLR4 signaling pathways in MEF cells, that it may upregulate IκBζ expression and that the high levels of IκBζ expression in ATF3-/- cells disrupts Kdo_2_-Lipid A-mediated signaling pathways.

## Introduction

Toll-like receptors (TLRs) are membrane-bound pattern-recognition receptors (PRRs) that sense a variety of microbial-specific motifs to trigger an innate immune response [1], [2]. To date, 13 mammalian TLRs have been identified, each of which detects specific ligands derived from invading microbes [3]. TLRs consist of an outward leucine-rich repeat (LRR) motif, a transmembrane domain and an inward Toll/interleukin-1 receptor (TIR) domain [4], [5]. TLRs are localized on the cytoplasmic or endosomal membrane, where they recognize diverse pattern-associated molecular patterns (PAMPs) such as bacterial lipopolysaccharide (LPS) or microbial nucleic acids through their LRR motifs [6]. Once a LRR motif is engaged by PAMPs, TLRs initiate specific signaling pathways via the recruitment of TIR domain-containing adaptor molecules such as myeloid differentiation primary response protein 88 (MyD88) or TIR domain-containing adaptor inducing IFN-β (TRIF) [7], [8].

TLR4 selectively recognizes LPS, a major outer membrane component of gram-negative bacteria, in cooperation with co-receptor MD2 [9]. LPS is composed of a lipid A portion that is responsible for its toxic properties, as well as a core polysaccharide and O-specific polysaccharide [10], [11]. Recently, the linkage of 3-deoxy-D-manno-octulosonic acid (Kdo), a component of the core polysaccharide, to lipid A was shown to be sufficient to induce the endotoxin activity of LPS [12]. Structural analyses have also revealed that the binding of LPS to TLR4-MD2 induces dimerization of the TLR4-MD2 complex, leading to activation of downstream signaling pathways [13]. Upon stimulation with LPS, TLR4 recruits MAL and TRIF through its TIR domain and then initiates a series of signaling cascades that result in the activation of nuclear factor kappa B (NF-κB) and mitogen-activated protein kinases (MAPKs) [14], [15].

Activating transcription factor 3 (ATF3) is a member of the ATF/cAMP response element binding-protein (CREB) family of basic-region leucine zipper (bZIP) type transcription factors. ATF3 binds to a cAMP response element sequence through its basic-region and forms homo- or heterodimers with other CREB family members through its bZip domain [16], [17]. ATF3 has been shown to repress or activate the transcription of target genes depending on the cell context [17], [18]. In contrast to the implication of its name, ATF3 is a homodimer that functions as a transcriptional activator [19]. ATF3 is a stress-response gene that is induced by a broad range of stimuli, including cytokines and physiological stresses [20]. In macrophages, ATF3 has been shown to be induced by the TLR4 ligand, LPS and bacillus Calmette Guerin (BCG), as well as by IFN α, β and γ in human PBMC [18]. Despite these observations, the role that ATF3 plays in innate immune responses has only been recently described [21]. Activation of the TLR signaling pathways has been shown to induce the expression of ATF3, which subsequently regulates the transcription of NF-κB-dependent genes such as IL-6 and IL-12β [21]. Studies involving ATF3-deficient mice have revealed that elevated cytokines were present in ATF3-/- mice upon stimulation with LPS, indicating that ATF3 negatively regulates the transcription of NF-κB-dependent genes [22]. It has also been shown that ATF3 is involved in regulation of the cell cycle and apoptosis; however, the biological role of ATF3 is still the subject of debate [23], [24].

In the present study, we investigated the function of ATF3 in the Kdo_2_-Lipid A-mediated TLR4 signaling pathways in mouse embryonic fibroblast (MEF) cells. We found that several genes were dramatically upregulated in wild type MEF cells upon Kdo_2_-Lipid A treatment and that they subsequently induced the TLR4 pathway, but that the treatment had little effect on these genes in ATF3 knockout cells. These findings suggest that ATF3 plays a role in TLR4-mediated gene expression. Additionally, our microarray data revealed that the basal level of IκBζ mRNA is relatively higher in ATF3-/- MEF cells, which was confirmed by the protein levels in these cells. Moreover, our results showed that high levels of IκBζ may inhibit Kdo_2_-Lipid A-mediated NF-κB activation in ATF3-/- MEF cells. We also demonstrated that Kdo_2_-Lipid A-induced activation of both NF-κB and JNK was affected in the absence of ATF3, but that the TNF-induced activation of NF-κB, JNK and cell death was normal. Thus, both NF-κB and JNK activation are regulated by ATF3 via the TLR4 signaling pathway. The negative feedback role of ATF3 for controlling cytokine toxicity provides an advantage as a model of infection with high clinical relevance. The role of ATF3 immune regulation and connections to infection associated diseases warrants further study.

## Results

### ATF3 is indispensable for Kdo_2_-Lipid A-induced NF-κB and JNK activation

**Figure 1:**
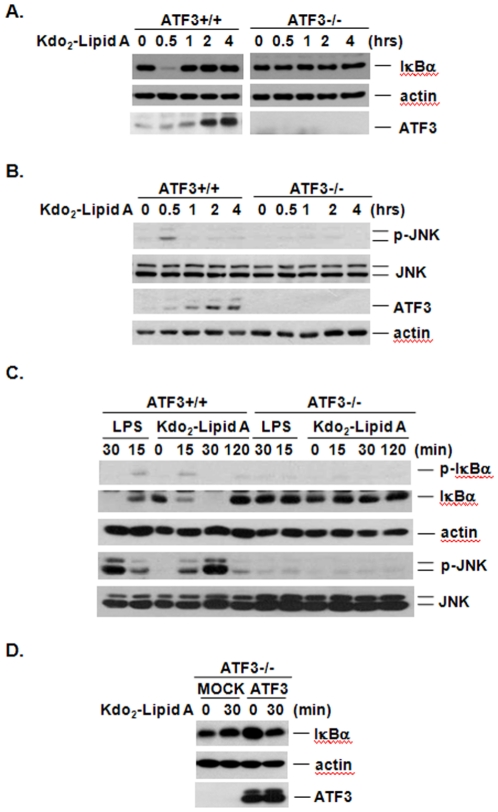
Loss of ATF3 caused defect of NF-κB and JNK activation upon Kdo_2_-Lipid A treatment in MEF cells. A. Wild type and ATF3-/- MEF cells (5×10^6^ cells) were treated with Kdo_2_-Lipid A (10 µg/ml) for the indicated times. Cell extracts were analyzed by immunoblotting with anti-IκBα, anti-ATF3 and anti-actin antibodies. B. The same cell lysates from A were analyzed by immunoblotting with anti-p-JNK, anti-JNK, anti-ATF3 and anti-actin antibodies. C. MEF cells were treated with 10 µg/ml of LPS or 10 µg/ml of Kdo_2_-Lipid A for the indicated times. Cell extracts were analyzed by immunoblotting. D. Western blots of ATF3-/- MEF cells transfected with mock or ATF3 plasmid and treated with Kdo_2_-Lipid A (10 µg/ml) for the indicated times. These experiments were repeated three times with similar results.

To gain insight into the biological role of ATF3 in TLR4-mediated signaling pathways, we treated MEF with Kdo_2_-Lipid A. Kdo_2_-Lipid A treatment caused degradation of IκB, which is an essential step in NF-κB activation that also leads to a significant increase in the cellular level of ATF3 in wild type MEF cells (Fig. 1A). Surprisingly, early degradation of IκBα was abrogated in ATF3-/- MEF cells that were treated with Kdo_2_-Lipid A, indicating that ATF3 is required for TLR4-dependent NF-κB activation (Fig. 1A). Next, we examined the phosphorylation of JNK in wild type and ATF3-/- MEF cells following Kdo_2_-Lipid A treatment. LPS stimulation was shown to activate the JNK signaling pathway in addition to NF-κB via the TLR4 signaling pathway. Similar to NF-κB activation, the Kdo_2_-Lipid A-induced phosphorylation of JNK was completely blocked in ATF3-/- MEF cells, whereas these pathways were potently activated in ATF3+/+ MEF cells at 30 min (Fig. 1B). To confirm these findings, we evaluated ATF3 to determine if it is critical for TLR-dependent NF-κB activation in response to LPS, which is a potent activator of TLR4-dependent NF-κB activation [14]. As shown in Figure 1C, both LPS and Kdo_2_-Lipid A induced phosphorylation of IκBα at 15 min and degradation of IκBα at 30 min in ATF3+/+ MEF cells, but not ATF3-/- MEF cells (see Fig. S1 for further information). We also confirmed that ATF3-dependent JNK activation in response to both LPS and Kdo_2_-Lipid A induced the TLR4 signaling pathway. As shown in Fig. 1C, both LPS and Kdo_2_-Lipid A treatment led to phosphorylation of JNK at 15–30 min in ATF3+/+ MEF cells, but not in ATF3-/- MEF cells, suggesting that Kdo_2_-Lipid A has the same effect as LPS, which is a known TLR4 activator. To further confirm that the inhibition of IκBα degradation in ATF3-/- MEF cells was the authentic effect of ATF3 deficiency, we examined the effect of ATF3 reconstitution in ATF3-/- MEF cells in response to Kdo_2_-Lipid A treatment. Transient transfection of ATF3 plasmid into ATF3-/- MEF cells restored the ATF3 protein levels and reconstitution of ATF3 induced IκBα degradation by Kdo_2_-Lipid A (Fig. 1D). Taken together, these data suggest that Kdo_2_-Lipid A-induced activation of both NF-κB and JNK through TLR4 signaling pathway requires ATF3 in MEF cells.

### ATF3 is dispensable for TNF-α-induced NF-κB and JNK activation

**Figure 2:**
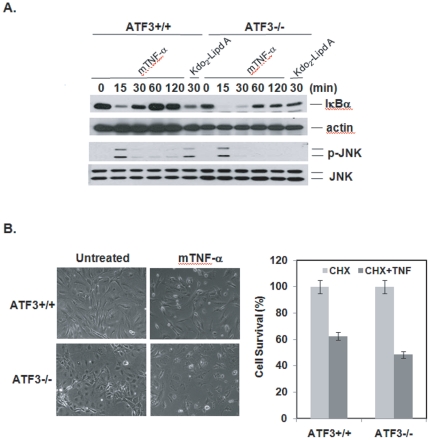
ATF3 is dispensable for TNF-α-induced NF-κB and JNK activation. A. Wild type and ATF3-/- MEF cells were treated with TNF-α (30 ng/ml) or Kdo_2_-Lipid A (10 µg/ml) for the indicated times, after which the cell extracts were analyzed by immunoblotting. Data are representative of at least three independent experiments. B. TNF-α induced cell death was measured by MTT assay. Cells were pretreated for 30 min with CHX (10 µg/ml) and then treated with TNF-α for 14 hours. The cell viability was then analyzed by MTT assay. Each data point represents the mean ± SEM of different experiments conducted under the same conditions. Left panel: representative images were taken by a phase-contrast microscope.

To rule out the possibility that deletion of ATF3 caused some defect in the receptor signaling pathways, MEF cells were treated with TNF-α to evaluate both NF-κB and JNK activation. TNF-α is a potent activator of both IκBα degradation and JNK phosphorylation to mediate inflammation and cell survival [25]. IκBα degradation and JNK phosphorylation were observed in both ATF3+/+ and ATF3-/- MEF cells upon stimulation with TNF-α, but only in ATF3+/+ cells following treatment with Kdo_2_-Lipid A (Fig. 2A). This observation demonstrates that ATF3 is indispensable for TLR4-mediated NF-κB and JNK activation. To further confirm that TNF-α-induced the signaling pathway in both ATF3+/+ and ATF3-/- MEF cells, we evaluated TNF-α-induced cell death. Regular MEF cells undergo apoptosis in response to treatment with the protein synthesis inhibitor, cycloheximide, 30 min before TNF-α treatment. As shown in Fig. 2B, both ATF3+/+ and ATF3-/- MEF cells were sensitive to TNF-α-induced apoptosis. Taken together, these results indicate that deletion of ATF3 disrupts TLR4 mediated signaling, but not TNFR1 in MEF cells.

### ATF3 deficiency causes a defect in Kdo2-Lipid A-induced gene transcription

**Figure 3:**
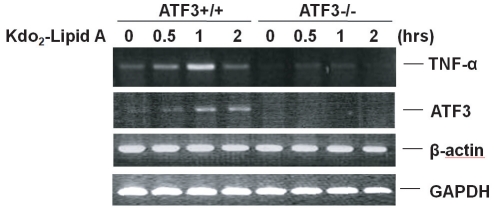
ATF3 deficiency causes a defect in Kdo_2_-Lipid A-induced gene transcription. Wild type and ATF3-/- MEF cells were treated with Kdo_2_-Lipid A (10 µg/ml) for the indicated times, after which the mRNA was isolated. The total relative levels of TNF-α and ATF3 mRNA were determined by RT-PCR. DNA bands were quantified. Data are representative of at least three independent experiments.

A common feature of signaling through TLR4 is that the induced expression of many of the pro-inflammatory cytokines is dependent on both NF-κB and MAP kinase-dependent transcription factors [26]. The results of the present study suggest that ATF3 deficiency blocked Kdo_2_-Lipid A-induced TLR4 signaling pathways in MEF cells. To further evaluate these findings, we investigated Kdo_2_-Lipid A-induced gene transcription to determine if it differed between wild type and ATF3-/- MEF cells using microarray analysis. To accomplish this, wild type and ATF3-/- MEF cells were treated with Kdo_2_-Lipid A for 0.5, 1 and 2 hrs, after which the total relative levels of TNF-α and ATF3 mRNA were determined by RT-PCR. As shown in Fig. 3, the relative levels of TNF-α and ATF3 increased upon Kdo_2_-Lipid A treatment in wild type MEF cells, but not in ATF3-/- MEF cells. β-actin and GAPDH were used as internal controls. These data suggest that Kdo_2_-Lipid A-mediated TLR4 pathways induce the expression of pro-inflammatory cytokines, and this was affected by ATF3 deficiency in MEF cells.

### ATF3 deficient MEF cells expressed a high level of IκBζ

**Figure 4:**
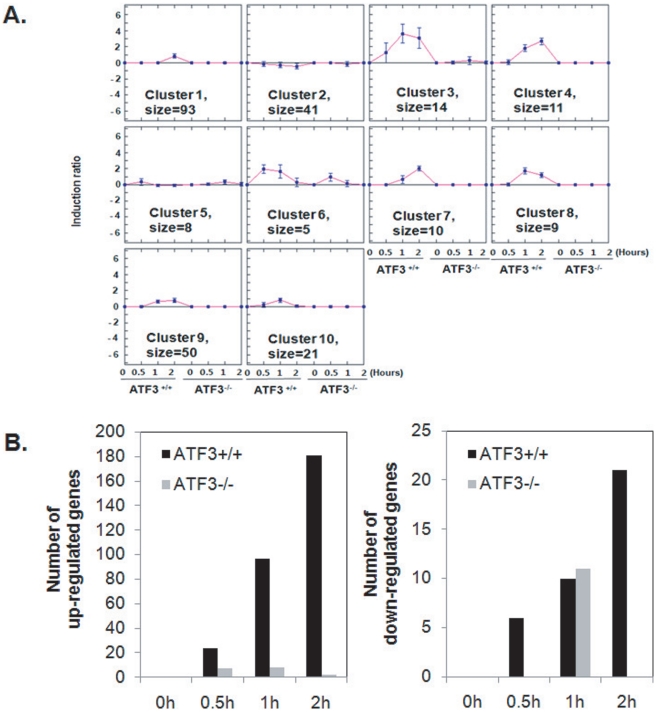
DNA microarray analysis of ATF3 MEF cells upon Kdo_2_-Lipid A treatment. A. Wild type MEF and ATF3-/- MEF cells were stimulated with 10 µg/ml Kdo_2_-Lipid A. The RNA was then isolated at the indicated times and subjected to DNA microarray analysis, after which the genes were clustered based on their kinetic profiles using a K-means algorithm and the average log_2_ (treated/control) values of normalized gene expression. B. Loss of the ATF3 gene induces distinct kinetic changes upon Kdo_2_-Lipid A treatment. The number of genes that showed significant changes (≥50%).

**Figure 5:**
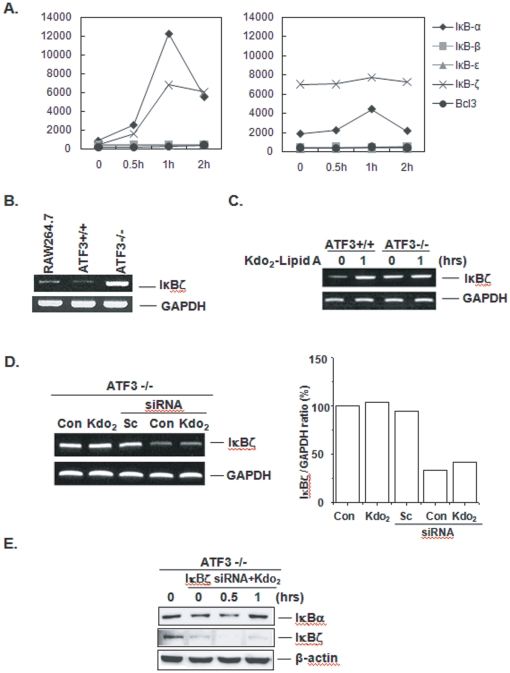
ATF3 deficient MEF cells expressed a high level of IκBζ. A. The gene expression patterns of the IκB family (α, β, ε, ζ and bcl-3) in ATF3+/+ (left) and ATF3-/- (right) MEF cells. B. Total relative levels of IκBζ mRNA were determined by RT-PCR in RAW 264.7, wild type and ATF3-/- MEF cells. C. Wild type and ATF3-/- MEF cells were treated with Kdo_2_-Lipid A for 1 hour, after which the mRNA was isolated. The total relative levels of IκBζ and GAPDH mRNA were determined by RT-PCR. D. Scramble (Sc; negative control) and IκBζ siRNA was applied to ATF3 -/- cells for 24 h, after which they were exposed to Kdo_2_-Lipid A for another 1 h. RT-PCR analysis of the IκBζ mRNA was conducted. E. IκBζ siRNA was applied into ATF3 -/- for 24 h and then exposed to Kdo_2_-Lipid A for the indicated time points, after which the cell lysates were applied for Western blot with the indicated antibodies. DNA bands were quantified. Data are representative of at least three independent experiments.

To evaluate the underlying mechanism of the role of ATF3, we examined the difference in gene expression between wild type and knockout ATF3 MEF cells using DNA microarray technology. Differential gene expression profiles obtained after Kdo_2_-Lipid A stimulation were clustered into groups of coregulated genes as shown in Fig. 4A. The gene expression patterns in ATF3+/+ and ATF3-/- cells that were treated with Kdo_2_-Lipid A were visualized using the MeV software. Based on a threshold of ≥1.5-fold increase in MEF, Kdo_2_-Lipid A upregulated 225 genes, 211 of which were upregulated only in ATF3+/+ cells and five (CCL7, Ch25h, Tnfaip3, Nfkbia, Cxcl1) of which were upregulated only in ATF3-/- cells (Fig. 4B and Fig. S2). Of these genes, Nfkbia (IκBα is a known transcriptional regulator. For these reason, we carefully analyzed the IκB proteins. As shown in Fig. 5A, the signal intensity of IκBζ in ATF3-/- cells was much higher than that of ATF3+/+ cells (up to sixteen-fold when compared to the WT basal level). However, after 1 hour of stimulation, the IκBζ levels in WT cells appeared to be the same as those in ATF3-/- cells, but the IκBζ levels of ATF3-/- MEF cells showed little change in response to Kdo_2_-Lipid A treatment. IκBα was upregulated upon treatment with Kdo_2_-Lipid A, which indicated that there was a feedback loop of NF-κB activation in wild type ATF3+/+ MEF cells, but only a slight change in ATF3-/- MEF cells (Table S1). To further investigate this phenomenon, the total relative levels of IκBζ mRNA were determined by RT-PCR in RAW 264.7, wild type and ATF3-/- MEF cells. As shown in Fig. 5B, ATF3-/- MEF cells had a higher level of IκBζ mRNA than macrophages or ATF3+/+ MEF cells, which is consistent with the microarray data. We also confirmed that the level of IκBζ mRNA was upregulated in ATF3+/+ MEF cells in response to Kdo_2_-Lipid A treatment, but that only slight changes were induced in ATF3-/- MEF cells (Fig. 5C).

To determine if increased IκBζ expression caused inhibition of TLR4-dependent NF-κB activation, we used siRNA to prevent IκBζ expression in ATF3-/- MEF cells. Application of IκBζ siRNA to ATF3-/- MEFs obviously reduced IκBζ mRNA levels significantly, as indicated by a 66% decrease (Fig. 5D). As shown in Figure 5E, the prevention of IκBζ expression led to IκBα degradation to Kdo_2_-Lipid A at 30 min, similar as in ATF3+/+ MEF cells. These findings indicate that upregulated IκBζ expression prevents IκBα degradation to Kdo_2_-Lipid A in MEF cells. Taken together, these data suggest that a deficiency of ATF3 led to an increase of IκBζ, and that this up-regulated IκBζ plays an inhibitory role in TLR4-mediated NF-κB activation.

**Figure 6:**
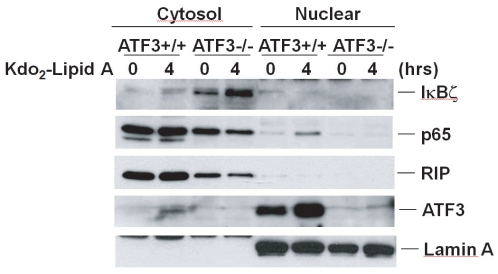
Abolished TLR4 activation in ATF3-/- MEF cells due to the up-regulation of IκBζ expression. ATF3-/- MEF cells had a high basal level of IκBζ expression. Wild type and ATF3-/- MEF cells were treated with Kdo_2_-Lipid A (10 µg/ml) for the indicated time and the cell extracts were then analyzed by immunoblotting with the indicated antibodies. These experiments were repeated three times with similar results.

Since our data suggested that there was a high level of IκBζ expression in ATF3-/- MEF cells, it is possible that deficiency of ATF3 caused the upregulation of IκBζ and translocation of IκBζ from the nuclear fraction into the cytosol to inhibit TLR4-mediated NF-κB activation. To test this possibility, we examined the localization of IκBζ by fractionation experiments. As shown in Figure 6, ATF3-/- MEF cells had a very high level of IκBζ expression in cytosol without Kdo_2_-Lipid A treatment, but wild type MEF cells had a very low level of IκBζ expression in cytosol. For the loading controls, we probed the samples with anti-RIP1 antibody for the cytosolic portion and anti-lamin A antibody for the nuclear portion. We also probed this blot with anti-ATF3 antibody to show the increase of ATF3 expression upon Kdo_2_-Lipid A treatment in ATF3+/+ MEFs. We confirmed that Kdo_2_-Lipid A induced NF-κB activation based on p65 translocation from the cytosol to the nucleus only in wild type MEF cells. These data suggest that ATF3 may function as a negative regulator of NF-κB and can inhibit the transcription of IκBζ in Kdo_2_-Lipid A-mediated TLR4 signaling. Furthermore, these results indicate that the deficiency of ATF3 up-regulates IκBζ expression in the cytosol as an inhibitor of NF-κB.

## Discussion

ATF3 is maintained at very low levels in quiescent cells, but is rapidly induced by various stresses, including UV, DNA damaging agents and LPS, indicating that many signaling pathways may be involved in the induction of ATF3 [27]. It was recently reported that ATF3 is a negative regulator of TLR4, which suggests that ATF3 regulates TLR4- stimulated inflammatory responses as part of a negative-feedback loop [28]. TLR4 recognizes LPS, which comprises the lipid A portion and triggers downstream signaling pathways [29], [30]. Upon stimulation with LPS, TLR4 initiates a series of signaling cascades that result in the activation of NF-κB and MAPKs. Chemically defined LPS, Kdo_2_-Lipid A, is fully active as an endotoxin based on various biological criteria and is highly selective for TLR4. In this study, we investigated the function of ATF3 in the Kdo_2_-Lipid A-mediated TLR4 signaling pathways in MEF cells.

As a TLR4 activator, Kdo_2_-Lipid A was found to have a bioactivity similar to LPS based on NF-κB activation and expression profiling of genes such as TNF-α, IL-1β and ATF3 in macrophages. We also showed that Kdo_2_-Lipid A initiates TLR4-mediated NF-κB and JNK activation in MEF cells. However, deficiency of ATF3 abolished TLR4-mediated NF-κB and JNK activation, as well as the patterns observed in the gene expression. The existing literature regarding the role of ATF3 in TLR4-mediated signaling suggests that ATF3 acts as a negative regulator in this pathway. Gilchrist et al. [28] demonstrated that a substantial increase in LPS-induced IL-6 and IL-12β mRNA levels in ATF3-/- bone-marrow derived macrophages and that LPS induction of iNOS and TNF mRNA was similarly enhanced in ATF3-/- mice. Conversely, ATF3 is required to activate TLR4-mediated NF-κB and JNK in cells such as macrophages, suggesting that ATF3 may also play a role in TLR signaling in a cell-type specific manner.

While TNF-induced phosphorylation of JNK and degradation of IκBα are normal in ATF3-/- MEF cells, Kdo_2_-Lipid A induced IκBα degradation and JNK phosphorylation are impaired in these cells. Therefore, deletion of ATF3 disrupts TLR4 mediated signaling, but not TNFR1 in MEF cells. Consistent with these findings, microarray analysis revealed that the most robust changes in transcription were induced by Kdo_2_-Lipid A in wild type MEF cells, but that few changes were induced in ATF3-/- MEF cells. Among the 225 genes that were upregulated in response to treatment with Kdo_2_-Lipid A, 211 genes were upregulated only in ATF3+/+ cells, while 5 genes were upregulated only in ATF3-/- cells.

One interesting gene that was significantly upregulated in ATF3-/- cells (up to sixteen-fold when compared to the basal level of ATF3+/+ cells) is IκBζ, which is an inducible nuclear protein that can selectively inhibit or activate certain NF-κB dimers [31]. Previous studies have suggested that IκBζ functions as an additional regulator of NF-κB activity and provides another level of control for the activation of NF-κB-dependent target genes [32]. IκBζ deficient mice show that IκBζ is essential for the expression of numerous LPS-inducible genes including IL-12, C/EBP-δ and GM-CSF [33]. In addition to its function as a positive regulator of NF-κB activation, it has been reported that the activity of NF-κB is controlled in a negative feedback loop by IκBζ expression. p65 and p50 are the major subtypes of NF-κB present in MEF cells and the mRNA expression level of p65 is five times higher than that of p50 in both ATF3+/+ and ATF3-/- MEF cells (Table S1). In this context, we have generated a structural model of IκBζ using molecular docking to search for potential sites of interaction between the IκBζ and p50/p65 heterodimer and the IκBζ and p50/p50 homodimer. The docking experiments revealed that the binding of IκBζ ankyrin repeats with the p50/p65 N-terminal DNA binding domain prevents NF-κB-mediated transcriptional activation (manuscript submitted separately). In line with its function as a negative regulator of NF-κB, we suggest that high levels of IκBζ expression in ATF3-/- cells may disrupt Kdo_2_-Lipid A-mediated signaling pathways.

It has been suggested that TLR4 leads to signal activation via downstream signaling factors such as MyD88, IRAKs and TRAF6, and that a TRAF6-dependent pathway engages MAPK and IKK, resulting in activation of transcription factors that participate in the induction of proinflammatory cytokines such as NF-κB and AP-1 [2], [34]. TRAF6 is essential for signal activation, as TRAF6 deficiency results in defective LPS signaling [35]. Wild type and ATF3-/- MEF cells were found to have similar levels of TRAF6 expression under unstimulated conditions and their expression levels were unchanged upon Kdo_2_-Lipid A treatment (data not shown), which suggests that the abolished NF-κB and JNK activation in response to Kdo_2_-Lipid A was not due to the TRAF6 expression in ATF3-/- MEF cells. The differential effect of ATF3 deletion on TLR4 signaling in MEF and macrophages was likely due to different levels of IκBζ. It has been suggested that IκBζ is induced in response to IL-1, LPS, peptidoglycan, bacterial lipoprotein, flagellin and CpG DNA, but not TNF-α. Furthermore, this nuclear protein interacts with NF-κB via its carboxyl-terminal ankyrin-repeats and can selectively inhibit or activate certain NF-κB dimers. For example, IκBζ provides transcription ability to p50 homodimers when complexed with them. Conversely, when complexed with p65-containing dimers, IκBζ can repress transcription [31], [36]. Consistent with microarray data and RT-PCR data, we can detect high levels of IκBζ expression in ATF3-/- MEF cells. These cells showed no NF-κB activation in response to Kdo_2_-Lipid A or after the prevention of IκBζ expression by knockdown led to IκBα degradation, which is similar to the results observed in ATF3+/+ MEF cells.

Taken together, these findings suggest that a deficiency of ATF3 led to an increase of IκBζ, and that this up-regulated IκBζ plays an inhibitory role in TLR4-mediated NF-κB activation. It is currently not known if the interference from NF-κB activation in ATF3-/- MEF cells is due to the high level of IκBζ expression or cellular localization of IκBζ. Since our data shows that IκBζ localized at the cytosol fraction and that the prevention of IκBζ expression by IκBζ specific-siRNA led to IκBα degradation to Kdo_2_-Lipid in ATF3-/- MEF cells, IκBζ may function as a negative regulator of the NF-κB pathway in TLR4 signaling in MEF cells. Despite the need to address the mechanisms discussed here in much greater detail and to provide a better understanding of the physiological functions of ATF3 in the TLR4-mediated pathway in MEF cells, our data suggest that ATF3 plays a role in Kdo_2_-Lipid A-mediated TLR4 signaling to activate NF-κB and MAP kinase, and that ATF3 is required for TLR4-dependent gene expression through the NF-κB pathway.

## Materials and Methods

### Reagents

Kdo_2_-Lipid A was purchased from Avantisis. Recombinant TNF-α and recombinant IL-1β were purchased from R&D Systems. Anti-ATF3, anti-TRAF6 and anti- IκB antibodies were obtained from Santa Cruz Biotechnology. Anti-actin antibody was purchased from Sigma and anti-p-IκB antibody was purchased from Cell Signaling. Anti-p-JNK antibody was purchased from Biosource and anti-JNK antibody was purchased from Phamingen. Anti-IκBζ antibody was obtained from Novus. LPS was purchased from Sigma and CHX was purchased from Calbiochem.

### Cell culture

Wild-type and ATF3-/- mouse embryonic fibroblast (MEF) cells were cultured as previously described [24]. MEF cells and RAW264.7 cells were cultured in Dulbecco's modified Eagle's medium supplemented with 10% fetal bovine serum, 2 mM glutamine, 100 U/ml penicillin and 100 µg/ml streptomycin. All animal procedures were conducted in accordance with the Sungkyunkwan University Guide for the Care and Use of Laboratory Animals.

### Western blot analysis

Protein analysis was conducted as previously described [37]. Briefly, MEF cells (5×10^5^ cells) were washed with PBS and resuspended in M2 lysis buffer (20 mM Tris at pH 7, 0.5% NP-40, 250 mM NaCl, 3 mM EDTA, 3 mM EGTA, 2 mM DTT, 0.5 mM PMSF, 20 mM β-glycerol phosphate, 1 mM sodium vanadate, 1 µg/ml leupeptin). Samples were vortexed for lysis at 4°C for 15 min and then centrifuged at 15,000×g for 5 min at 4°C. The samples were then heated at 95°C for 5 min, after which they were briefly cooled on ice. Following centrifugation at 15,000×g for 5 min, 50 µg of the cell lysates were fractionated by SDS-polyacrylamide gel electrophoresis on a 12% gel. Resolved proteins were transferred overnight to polyvinylidene difluoride membrane in 25 mM Tris, pH 8.5, 0.2 mM glycerin, 20% methanol at 25 V. Blots were blocked for at least 2 h with TBST containing 10% nonfat dry milk. The proteins were visualized by enhanced chemiluminescence (ECL) according to the manufacturer's instructions (Amersham).

### Nuclear extraction

Cell lysates of wild type and ATF3/- MEF cells stimulated for the indicated times were prepared in NP40 lysis buffer supplemented with 1 mg/ml each of leupeptin and aprotinin and 1 mM phenyl methylsulphonyl fluoride (PMSF). After centrifugation at 12,000 rpm for 15 min, the protein concentrations of the lysates were determined using a Bio-Rad protein assay kit. To separate the cytoplasmic and nuclear fractions, cell pellets were processed using a NE-PER Nuclear and Cytoplasmic Extraction kit (Thermo Fisher Scientific Inc.) according to the manufacturer's instructions.

### RNA extraction and RT-PCR

Total RNA was isolated from MEFs according to the manufacturer's instructions using a RNeasy Mini Kit (Qiagen Inc.). The total relative levels of TNF-α, ATF3 and IκBζ mRNA were determined by RT-PCR using amfiSure PCR Premix (GenDePOT). PCR amplification was conducted using Taq DNA polymerase in 30 amplification cycles for TNF-α, IκBζ, β-actin and GAPDH (95°C for 1 min, 60°C for 1 min, and 72°C for 1.5 min). The PCR primers were as follows: ATF3- F; 5′-ATG ATG CTT CAA CAT CCA GGC-3′ and R; 5′-TTA GCT CTG CAA TGT TCC TTC-3′ [38]; TNF-α- F; 5′-AGC CCC CAG TCT GTA TCC TT-3 and R; 5′-CTC CCT TTG CAG AAC TCA GG-3′ [39]; β-actin- F; 5′-TTC TTT GCA GCT CCT TCG TTG CCG-3′ and R; 5′-TGG ATG GCT ACG TAC ATG GCT GGG-3′ [27]; GAPDH: F; 5′-CAT GTA GGC CAT GAG GTC CAC CAC-3′ and R; 5′-TGA AGC AGG CAT CTG AGG G-3′ [40]; IκBζ- F; 5′-ACA TCA CCG CAA ACG CCT ACA CCT G-3′ and R; 5′-CGT GTC ACC ATC TCC GTC CCT AGC G-3′ [41]. The final PCR products were resolved in 1.5% agarose gel and stained with ethidium bromide.

### DNA microarray

Wild type MEF and ATF3-/- cells were stimulated with Kdo_2_ -Lipid A (10 ug/ml) for 30 minutes, 1 hour or 2 hours. RNA samples were prepared for microarray analysis using a RNeasy Mini Kit (Qiagen Inc.) according to the manufacturer's protocols. Microarray analysis was conducted using the Illumina Bead Chip. Briefly, 500 ng of total RNA were used to conduct in vitro transcription amplification with the Illumina RNA amplification kit (Ambion). The amplified RNA was then hybridized to Mouse-Ref-v2 Expression Bead Chips containing gene-specific oligonucleotides (∼22,000 genes, Illumina, Inc., San Diego, CA) [42]. Hybridization was detected using 1 µg/ml of Cy3-steptavidine (Amersham Biosciences), after which the chips were scanned with an Illumina BeadArray Reader. Image analysis was conducted using Illumina's Bead Studio software, and the raw data were exported into Microsoft Access [43]. Quantile normalized data were selected based on a p-value <0.05 and a fold change of at least 1.5.

### Microarray data analysis

The scanned data were initially analyzed using the Illumina BeadStudio software. Intensity values derived from the hybridization method were used, and probes that had a p-value <0.05 were selected for further analysis. The genes that showed higher (≥50% change) or lower expression (≥50% change) in response to treatment were categorized as upregulated or downregulated genes, respectively. The log_2_ ratio value was hierarchically clustered to visualize the patterns of gene expression change using Multiple Experiment Viewer (www.tigr.org/software/tm4/mev.html).

### IκBζ knockdown

siRNAs targeting IκBζ mRNA were designed and purchased from Genolution Pharmaceuticals (Seoul, Korea). Transfection was conducted using Lipofectamine 2000 (Invitrogen) according to the manufacturer's instructions. To examine the effect of siRNA, B16F10 cells were co-transfected with 10 nM siRNA. The cells were harvested 24 h after transfection, and the potency of siRNA to silence IκBζ expression was measured by RT-PCR using the forward primer: 5′-CAG TAT CGG GTG ACA CAG TTG G- 3′, and the reverse primer: 5′-GGA TAC GTC GGA TCT GTT CTC C- 3′. The siRNAs showing efficient IκBζ silencing in B16F10 cells were selected and each was transfected (30 nM) to ATF3 -/- MEF cells. Scramble siRNA purchased from Genolution Pharmaceuticals was applied to control cells as a negative control. After culturing ATF3 -/- cells in medium for 24 h, siRNA solution, which was diluted in a siRNA transfection medium, was added to the ATF3 -/- cells. Following transfection with scrambled or IκBζ siRNA for 24 h, the medium was replaced with normal medium, and ATF3 -/- cells were treated with Kdo_2_-Lipid A.

### Cytotoxicity assay

TNF-induced cell death was determined using a colorimetric 3-[4,5-dimetylthiazol-2-yl]-2,5-diphenyltetrazolium bromide (MTT) assay. MEF cells (2×10^5^ cells) were cultured in 4-well plates for 14 h after treatment with TNF-α (30 ng/ml). Next, 20 µl of MTT solution (5 mg/ml) were added and the cells were incubated at 37°C for an additional 4 h. After washing the supernatant out, the insoluble formazan product was dissolved in DMSO. The optical density of the 96-well culture plates was then measured at 570 nm using an ELISA reader. The optical density of formazan formed in untreated control cells was taken to indicate 100% viability.

### Statistical analysis

Statistical analysis was conducted using the Illumina BeadStudio software. The data represents the mean and standard error of the mean (SEM) of different experiments under the same conditions. All results shown are representative of three experiments that were conducted independently. Treated groups and the controls were compared using a Student's *t*-test and a *p*<0.05 was considered significant.

## Supporting Information

Figure S1 Both LPS and Kdo_2_-Lipid A induced NF-κB activation in ATF3+/+ MEF cells, but not in ATF3-/- MEF cells. Wild type and ATF3-/- MEF cells were treated with either LPS (10 µg/ml) or Kdo_2_-Lipid A (10 µg/ml) for 30 min. Cell lysates were applied for the Western blot with the indicated antibodies to show that both TLR4 activators had the same effect on NF-κB activation.
(0.14 MB DOC)

Figure S2 DNA microarray analysis of ATF3 MEF cells upon Kdo_2_-Lipid A treatment. The dendrogram shows hierarchically clustered transcriptional changes induced by treatment with Kdo_2_-Lipid A in wild type and ATF3-/- MEF cells for the indicated times.
(0.31 MB DOC)

Table S1 Gene expression profile of the IκB and NF-κB families. The numbers indicate the relative hybridization intensities observed upon analysis by an Illumina Bead Array.
(0.04 MB DOC)
