# Supplementary material for: ATF3 Plays a Key Role in Kdo2-Lipid A-Induced TLR4-Dependent Gene Expression via NF-κB Activation
Source: PLoS One. 2010 Dec 2;5(12):e14181. doi: 10.1371/journal.pone.0014181 (PMC2996292; doi:10.1371/journal.pone.0014181)
Supplement: Figure S1 — Both LPS and Kdo2-Lipid A induced NF-κB activation in ATF3+/+ MEF cells, but not in ATF3-/- MEF cells. Wild type and ATF3-/- MEF cells were treated with either LPS (10 µg/ml) or Kdo2-Lipid A (10 µg/ml) for 30 min. Cell lysates were applied for the Western blot with the indicated antibodies to show that both TLR4 activators had the same effect on NF-κB activation. (0.14 MB DOC) [file pone.0014181.s001.doc]

**Supporting Information File #1**

**Fig. S1. *Both LPS and Kdo_2_-Lipid A induced NF-κB activation in ATF3+/+ MEF cells, but not in ATF3-/- MEF cells.*** Wild type and ATF3-/- MEF cells were treated with either LPS (10 µg/ml) or Kdo_2_-Lipid A (10 µg/ml) for 30 min. Cell lysates were applied for the Western blot with the indicated antibodies to show that both TLR4 activators had the same effect on NF-κB activation.

**Fig. S1.**
