# Supplementary material for: ATF3 Plays a Key Role in Kdo2-Lipid A-Induced TLR4-Dependent Gene Expression via NF-κB Activation
Source: PLoS One. 2010 Dec 2;5(12):e14181. doi: 10.1371/journal.pone.0014181 (PMC2996292; doi:10.1371/journal.pone.0014181)
Supplement: Figure S2 — DNA microarray analysis of ATF3 MEF cells upon Kdo2-Lipid A treatment. The dendrogram shows hierarchically clustered transcriptional changes induced by treatment with Kdo2-Lipid A in wild type and ATF3-/- MEF cells for the indicated times. (0.31 MB DOC) [file pone.0014181.s002.doc]

**Supporting Information File #2**

**Fig. S2. *DNA microarray analysis of ATF3 MEF cells upon Kdo_2_-Lipid A treatment.***

The dendrogram shows hierarchically clustered transcriptional changes induced by treatment with Kdo_2_-Lipid A in wild type and ATF3-/- MEF cells for the indicated times.

**Fig. S1.**
