# Supplementary material for: ATF3 Plays a Key Role in Kdo2-Lipid A-Induced TLR4-Dependent Gene Expression via NF-κB Activation
Source: PLoS One. 2010 Dec 2;5(12):e14181. doi: 10.1371/journal.pone.0014181 (PMC2996292; doi:10.1371/journal.pone.0014181)
Supplement: Table S1 — Gene expression profile of the IκB and NF-κB families. The numbers indicate the relative hybridization intensities observed upon analysis by an Illumina Bead Array. (0.04 MB DOC) [file pone.0014181.s003.doc]

**Supporting Information File #3**

**Table S1. *Gene expression profile of the IκB and NF-κB families.*** The numbers indicate the relative hybridization intensities observed upon analysis by an Illumina Bead Array.
